# Biodegradation of Microplastic Derived from Poly(ethylene terephthalate) with Bacterial Whole-Cell Biocatalysts

**DOI:** 10.3390/polym10121326

**Published:** 2018-11-30

**Authors:** Jixian Gong, Tongtong Kong, Yuqiang Li, Qiujin Li, Zheng Li, Jianfei Zhang

**Affiliations:** 1 School of Textiles, Tianjin Polytechnic University, No. 399 Bin Shui Xi Road, Xi Qing District, Tianjin 300387, China; kttcuz@163.com (T.K.); vicmaldini@126.com (Q.L.); lizheng_nx@163.com (Z.L.); zhangjianfei1960@outlook.com (J.Z.); 2 Collaborative Innovation Center for Eco-Textiles of Shandong Province, 308 Ningxia Road, Qingdao 266071, China; 3 Transfar Zhilian Co., Ltd., Hangzhou 311215, China; lyq1531@126.com

**Keywords:** microplastic, biodegradation, poly(ethylene terephthalate), whole-cell, combinatorial processing

## Abstract

At present, the pollution of microplastic directly threatens ecology, food safety and even human health. Polyethylene terephthalate (PET) is one of the most common of microplastics. In this study, the micro-size PET particles were employed as analog of microplastic. The engineered strain, which can growth with PET as sole carbon source, was used as biocatalyst for biodegradation of PET particles. A combinatorial processing based on whole-cell biocatalysts was constructed for biodegradation of PET. Compared with enzymes, the products can be used by strain growth and do not accumulated in culture solution. Thus, feedback inhibition of products can be avoided. When PET was treated with the alkaline strain under high pH conditions, the product concentration was higher and the size of PET particles decreased dramatically than that of the biocatalyst under neutral conditions. This shows that the method of combined processing of alkali and organisms is more efficient for biodegradation of PET. The novel approach of combinatorial processing of PET based on whole-cell biocatalysis provides an attractive avenue for the biodegradation of micplastics.

## 1. Introduction

In recent years, the pollution of microplastic has raised increasing concerns worldwide and is now an emerging research area [1]. Microplastics were defined as plastic particles <5 mm in size [2], which can be formed from the degradation of larger plastic consumer products and the production of plastic debris in industrial processes. It was found that fibers and fragments were the most common type of microplastic [3]. The fragments appear to have resulted from degradation of larger items. The fibers may be derived from synthetic clothing and fabrics which would release microfibers when they were worn and washed [4]. Microplastics may enter the receiving waters, such as brooks, rivers, seas and oceans, through multiple pathways, including stormwater runoff, wind advection and atmospheric fallout, and treated wastewater discharges [5]. Nowadays, microscopic plastic fragments and fibers have been found in coastal surface sediments, the pelagic zone, fresh water, soil and even tap water, bottled water, beer sugar and salt [6,7,8,9,10,11,12]. Microplastics can be up-taken by a wide range of marine organisms by different processes [13,14,15]. Ingestion of microplastic provides a potential pathway for the transfer of pollutants, monomers, and plastic-additives to organisms with uncertain consequences for their health. And then the microplastic could be accumulated in higher species, including man. So, the contamination of environment by microplastics is of concern not only because of the ecological impacts but also because they may compromise food security, food safety and consequently human health.

It was reported that a large proportion of microplastic fibers found in the marine environment may be derived from sewage as a consequence of rinsing of synthetic textiles [16]. Polyethylene terephthalate (PET) fiber generally referred to as polyester in the textile industry is the most widely used synthetic fiber, which consumes the majority of the PET produced globally. Owing to its excellent physical and chemical properties, PET has also been widely used in other fields as packaging materials, beverage bottles and functional material. However, the large amounts of PET that enters and accumulates in the ecosystem poses a great environmental challenge.

Although Polyethylene terephthalate has been previously considered as recalcitrant to biological degradation, PET-hydrolyzing enzymes exhibiting hydrolytic activity against PET films, fibers and fabrics have been reported [17,18,19,20,21,22,23,24,25,26]. Recently, Yoshida et al. [27] isolated a novel bacterium, Ideonella sakaiensis 201-F6, which is able to use PET as its major energy and carbon source, and convert PET into its two environmentally benign monomers. This provide an eco-friendly solution to PET accumulation in the environment. Functionalization of polyester can be achieved by bioprocessing with enzymes [28,29,30,31,32,33,34]. However, the application of enzymes as catalysts in the biodegradation of contamination usually involves a time-consuming and costly preparation process. The use of microorganisms as whole-cell biocatalysts [35,36,37,38,39] avoids the laborious production and purification steps in production of enzymes and allows a repeated use in sequential reactions. The enzymes can be protected within cellular environment from destabilizing and degrading effects which is problematic in case purified enzymes are used when the pH and temperature of the treatment fluid change. Moreover, the cell of microorganisms comprises a broad variety of other enzymes which might interfere with the desired reaction. Thus, the use of microorganisms as whole-cell biocatalysts provides a promising alternative for biodegradation of recalcitrant contamination.

In our previous study, a kind of aeromonas strain, Comamonas testosterone F4, was isolated from the waste water of PET production factory and applied in biodegradation of PET fibers [40]. Then this strain was improved and the engineered strain F5 was obtained by evolutionary engineering [41], which can grow with PET particles as sole carbon source under the alkaline conditions of pH = 11. Last, the strain F5 was further improved to be an alkali tolerant bacterium, Comamonas testosterone F6, which was grown in a treatment solution of pH = 12. This provides a possibility for biodegradation of microplastic derived from PET in a green route with biocatalysts. 

In this study, the micro-size particles were employed as substrate to simulate the biodegradation of microplastic. A combinatorial processing based on whole-cell biocatalysts was constructed for biodegradation of PET particles by Comamonas testosterone F6. The catalytic performance of whole-cell biocatalysts and synergy behavior on biodegradation of PET particles were estimated through detection of products of PET particles in bioprocess, measurement of the bacteria growth and its utilization of decomposition product, and characterization of PET particles after biodegradation.

## 2. Materials and Methods

### 2.1. Microorganism

C.testosteroni F4 and Alkali-resistant testosterone F6 were screened and cultivated by The Ecology Dyeing and Finishing Group of the Textile College of Tianjin Polytechnic University.

### 2.2. Materials

PET (Tianjin University of Technology Research Institute, Tianjin, China); terephthalic acid (TA), benzoic acid (BA), methyl acrylate (MA) and mono-(2-hydroxyethyl) terephthalate (MHET), bis-(2-hydroxyethyl) terephthalate (BHET) (Tianjin Yingchen Biotechnology Development Co., Ltd., Tianjin, China); H_3_PO_4_, CH_3_OH (Tianjin Komiou Chemical Reagent Co., Ltd., Tianjin, China); NH_4_Cl (Tianjin Jiangtian Chemical Co., Ltd., Tianjin, China); KH_2_PO_4_, Na_2_HPO_4_, MgSO_4_·7H_2_O, H_3_BO_3_, FeCl_3_·6H_2_O, MnSO_4_·5H_2_O, ZnCl_2_, CuSO_4_·5H_2_O (Tianjin Sailboat Chemical Reagent Technology Co., Ltd., Tianjin, China); NaCl (Tianjin Pharmaceutical Company, Tianjin, China); (NH4)_6_Mo_7_O_24_·7H_2_O (Tianjin Chemical Reagent, Tianjin, China).

### 2.3. Preparation of PET Particles

A certain number of PET slices were dried at a constant temperature of 150 °C for 12 h, and placed in a certain mode of a Chinese medicine pulverizer (Weinengda Electric Co., Ltd., Lanxi, China) for 2 h. The pulverized PET powders were sieved with a standard mesh of 500 mesh and 2000 mesh to obtain PET particles having a particle diameter of less than 10 μm. Wash PET samples three times with sterile saline before testing to remove the impurities adsorbed thereon.

### 2.4. Degradation Experiment

The medium was prepared according to Table 1 and Table 2. 100 mL of the medium was placed in a Erlenmeyer flask (500 mL). After sterilization, 20 mL of the starting strain culture solution was transferred, and then cultured at 37 °C and 140 rpm/min on a shaker (HZQ-Q full temperature oscillator, Harbin Donglian Electronic Technology Development Co., Ltd., Harbin, China), and periodically sampled and tested.

### 2.5. Characterization

#### 2.5.1. Detection of Strain Biomass

The biomass of the bacterial liquid was represented by the optical density (OD density) of the microbial fermentation broth measured by a V-1200 spectrophotometer (Shanghai Meipuda Instrument Co., Ltd., Shanghai, China). 3 mL of the strain culture solution was placed in the cuvette at regular intervals and the absorbance was measured at λ = 600 nm.

#### 2.5.2. Laser Particle Size Scattering Instrument

The average particle diameter (diameter d) and particle size distribution of the PET nanoparticles were determined by a dynamic light scattering method using a laser particle size scattering instrument (LPS, ZetaSizer Nano-Series, Malvern, UK). The 1.5 mL sample, which was not diluted, was placed in a plastic cuvette and placed in a card slot. The software was started for testing. Each sample was tested three times and the average was used for statistical analysis.

#### 2.5.3. Scanning Electron Microscopy (SEM)

The surface morphology and dimensions of the samples were characterized by TM3030 scanning electron microscopy (SEM, Hitachi, Tokyo, Japan) at an accelerating voltage of 20 kV.

#### 2.5.4. Differential Scanning Calorimetry (DSC)

The thermal properties of PET particles were tested and analyzed by differential scanning calorimetry (DSC, DSC200F3, Netzsch, Bavaria, Germany). The temperature was raised from room temperature to 300 °C at a rate of 10 °C/min. The first heating curve was tested and the thermal properties of the experimental samples were observed. Crystallinity of PET sample was calculated from the following Equation (1) is:

*X*(%) = [(Δ*H*_m_ − Δ*H*_c_)/Δ*H*_m_°] × 100% (1)

where Δ*H*_m_ and Δ*H*_c_ indicate the melting enthalpy (J/g) and the cold crystallization peak enthalpy (J/g), respectively, which were calculated from the fusion peak in DSC curve; Δ*H*_m_° represents the melting enthalpy of the 100% crystallization of the experimental sample, and the PET is 140 J/g.

#### 2.5.5. Reversed-Phase High Performance Liquid Chromatography (RP-HPLC)

The equipment used was a Essentia LC-15C pump (Shimadzu Corporation, Kyoto, Japan). The degradation products of PET were separated using an RP-C18/C8 (250 mm × 4.6 mm) at a flow rate of 1 mL/min on a purifier system equipped with a manual sample injector and SPD-15C UV visible dual wavelength detector. A mixture of 60% methanol and 40% water (50 mmolL^−1^ KH_2_PO_4_, H_3_PO_4_, pH = 2) (*v*/*v*) was used as the mobile phase. The injection volume was 5 μL and the column was maintained at a temperature of 40 °C. Separated products were detected at a wavelength of 240 nm. Standard curves of TA, MA and BHET were prepared for the conversion of standard products peak area to concentration.

## 3. Results and Discussion

### 3.1. Products of PET in Whole-Cell Biodegradation

In order to analyze the treatment effect of the PET combination processing method, the PET particles were placed in a neutral medium containing PET-decomposing bacteria F4, an alkaline medium containing an alkali-resistant PET-decomposing bacteria F6, and an alkaline medium containing no bacteria. After 48 h of culture, the PET decomposition products in the fermentation broth were examined.

**Figure 1:**
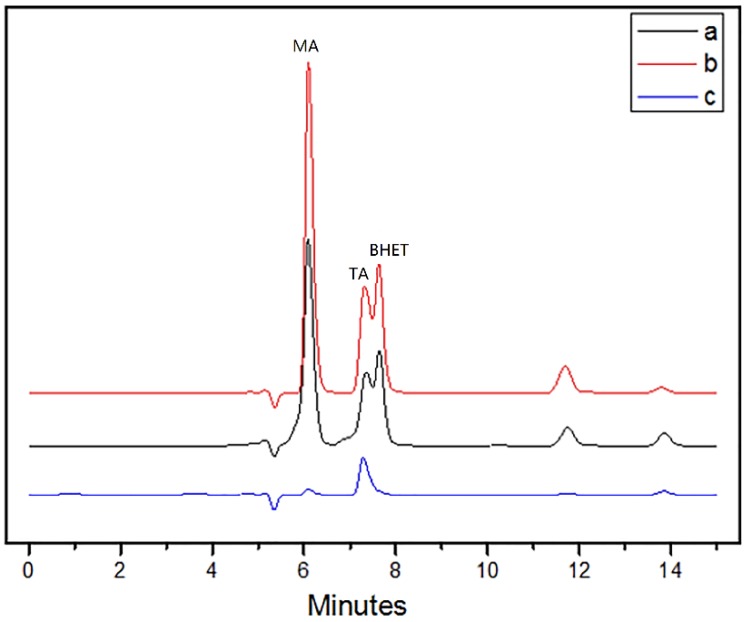
HPLC chromatogram of PET culture solution in whole cell catalysis. (**a**) whole-cell biocatalyzed PET decomposition products under neutral conditions. (**b**) whole-cell biocatalyzed PET decomposition products under alkaline conditions. (**c**) PET decomposition products under alkaline conditions.

As shown in Figure 1, the decomposition product of PET under the condition of alkali catalysis was relatively simple, mainly TA. In the biocatalytic conditions of PET decomposition, the products were more complicated which contained BHET, MHET, MA and TA. Under the two different conditions of alkali-resistant PET decomposition bacteria F6 and PET decomposition bacteria F4, the types of PET decomposition products were the same, only the difference in quantity. Alkali-resistant strains had better decomposition effects on PET under alkaline conditions. 

Comparing the amount of PET decomposition products under different conditions, it was found that the amount of biocatalytic PET decomposition products was the highest under alkaline conditions. In the case of biodegradation under alkaline conditions, the effect of biodegradation should be dominant because the amount of PET base hydrolysate was small. Comparing the biodegradation under alkaline conditions and neutral conditions, it was found that the amount of biodegradation products under alkaline conditions was much higher than that under neutral conditions. Moreover, the amount of the biodegradation product under alkaline conditions was greater than the sum of the amount of biodegradation under neutral conditions and the amount of alkali hydrolysis. This shows that the biocatalytic decomposition effect of PET under alkaline conditions is not only a simple superposition of biocatalysis and base-catalyzed hydrolysis.

### 3.2. Growth of Bacteria and Its Utilization of Decomposition Product

**Figure 2:**
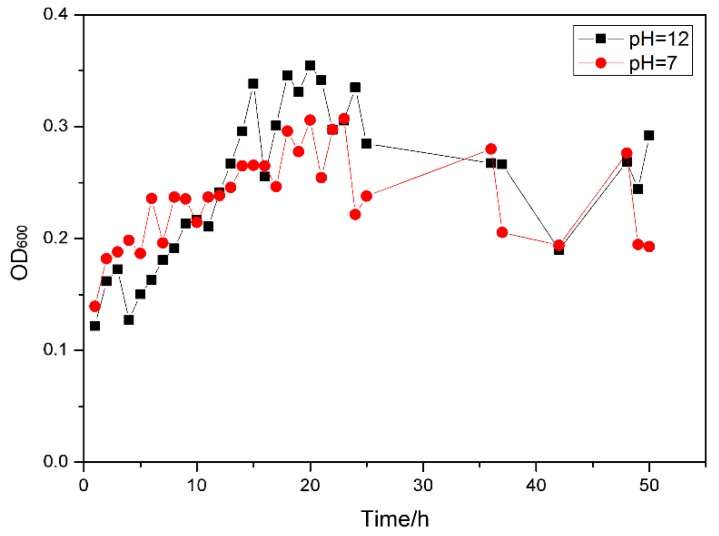
Biomass of strains using PET as a carbon source.

The neutral PET decomposing bacteria F4 and the alkali-resistant PET decomposing bacteria F6 were respectively cultured for 48 h under the conditions of pH = 7 and pH = 12 with PET microparticles as the sole carbon source. The growth of the strain is shown in Figure 2.

In this study, PET microparticles were the only carbon source for strain growth, and there were no other carbon sources in the medium. It is speculated that the growth of microorganisms utilizes a soluble small molecular substance formed by decomposition of PET as a carbon source. Studies have shown that PET can form small molecules such as TA, BHET, MHET, and BA after decomposition. However, the formation and presence of products in whole cell biocatalytic systems are different compared to enzymatic degradation of PET processes. The PET bioprocessing process using the enzyme directly as a biocatalyst, with the decomposition of PET, the accumulation of products such as TA whose concentration is used as an indicator to characterize the degree of decomposition of PET; While in the whole cell biocatalysis process, the decomposition products of PET such as TA in the fermentation broth do not accumulate and its concentrations fluctuate. This indicates that small molecules produced by decomposition of PET during fermentation of the strain will be utilized by the strain.

**Figure 3:**
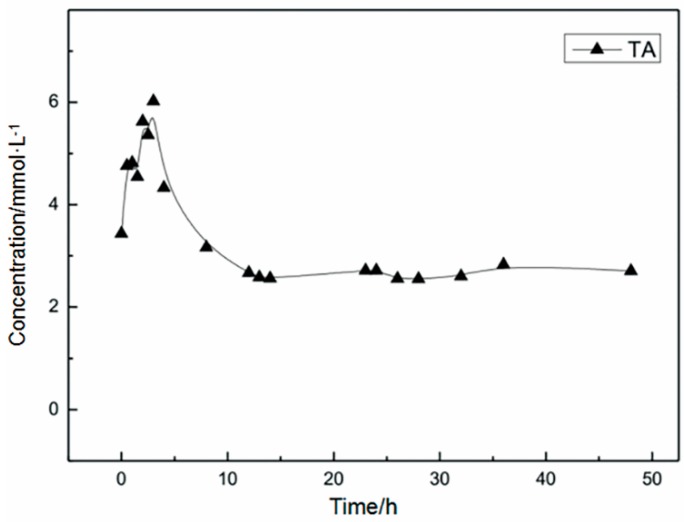
Utilization of TA by strains.

In order to investigate the ability of PET-decomposing bacteria to utilize the products of PET degradation, the concentration change of the product TA was monitored, and the results are shown Figure 3.

As shown in Figure 3, the yield of the product TA increased with time and then slowly decreased to a certain equilibrium state, which demonstrated that in whole-cell biocatalysis, small molecules produced by PET decomposition can be taken up by cells for metabolism. Studies have shown that PET decomposition products, especially MHET, also inhibit PET degrading enzymes. In PET bioprocessing with enzymes as biocatalysts, the inhibition of decomposition products will ultimately affect the effect of PET degrading enzymes. However, in the whole cell biocatalytic process, MHET will be further decomposed and utilized and the acidic products TA and BA will also be neutralized by the base without feedback inhibition of the product.

### 3.3. Characterization of PET Particle after Biodegradation

#### 3.3.1. Morphological Structure of PET Substrate

**Figure 4:**
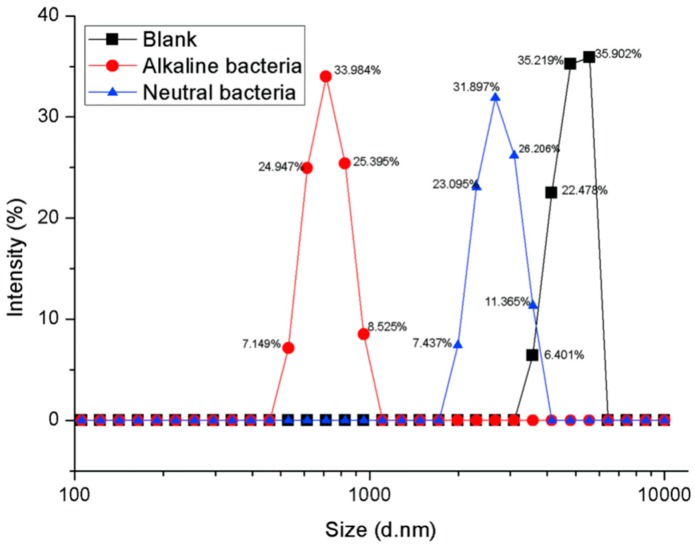
Changes in particle size of PET substrate after whole cell biological treatment under different pH conditions (Intensity %: the percentage of particles of different particle sizes to total particles).

The PET particles with an average particle size of 7.3 um were treated with PET-decomposing bacteria F4 under the condition of pH = 7 and alkali-resistant PET decomposing bacteria F6 and pH = 12. The particle size distribution of the PET substrate after the detection treatment is shown in Figure 4.

The average particle diameters of the untreated, neutral bacteria-treated and alkali-resistant bacteria-treated PET particles were tested to be 7.3, 2.63, and 1.58 μm, respectively. It can be seen from the experimental results that the average particle diameter of the PET substrate became small, and the average particle diameter was changed from 7.3 to 1.58 and 2.63 μm after biologically treated with the whole cell as a biocatalyst. This indicates that there is surface erosion in the biodegradation process of PET, that is, the decomposition of the polymer substrate starts from the surface, which was first discovered in biodegradation with membrane as a substrate.

In the present study, although the average particle diameter of the substrate PET particles was on the order of micrometers, a small number of nanoscale particles were also present. It is speculated that smaller particles may be preferentially degraded because the nanoscale particles enter the cells under endocytosis when interacting directly with the cells. If the case exists in whole-cell biocatalytic process of PET, then the last remaining should be larger particles, and the average particle size of the remaining PET detected should be larger. However, the experimental results show that the average particle size of the biological substrate becomes smaller after biological treatment, which indicates that the biodegradation mechanism of PET particles still starts from surface erosion.

**Figure 5:**
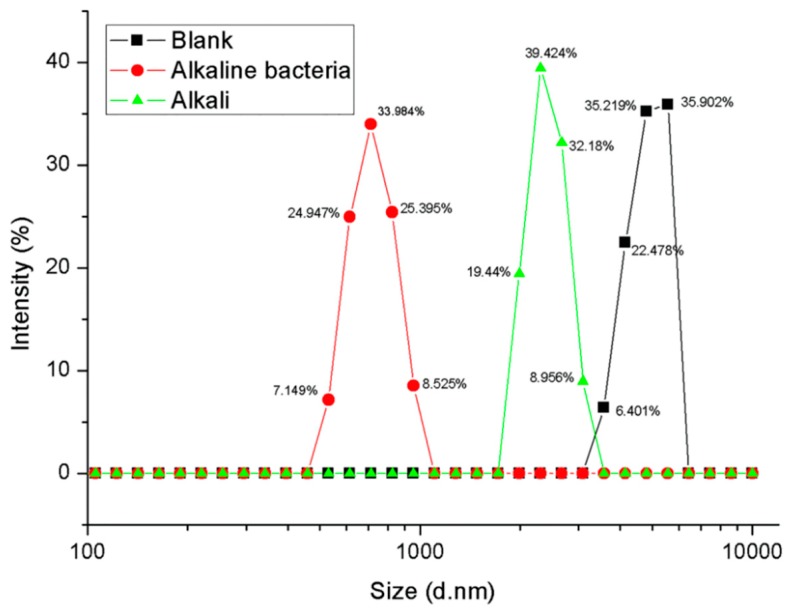
Changes in particle size of PET particles treated by alkali-resistant strain and alkali (Intensity %: the percentage of particles of different particle sizes to total particles).

It was also found that the average particle diameter of the PET substrate treated under alkaline conditions was smaller than that under neutral conditions. This indicates that biodegradation under alkaline conditions is more efficient than under neutral conditions. The decomposition of alkaline conditions PET should be the result of a combination of biodegradation and alkaline hydrolysis, not just the effect of alkaline hydrolysis, although alkaline hydrolysis of PET is also typically initiated from the surface. To confirm this, the PET particles were separately treated with an alkali-resistant strain and a base, and the results are shown in Figure 5.

The average particle diameters of the untreated, alkaline buffer-treated and alkali-resistant bacteria-treated PET particles were tested to be 7.3, 3.86, and 1.58 μm, respectively. As shown in Figure 5, the average particle diameters of PET particles were changed from 7.3 to 1.58 and 3.86 μm after treated by the alkali-resistant strain and the alkali. The result indicates that the biodegradation of PET under alkaline conditions should be the result of the combination of biodegradation and alkali hydrolysis, and the effect of biodegradation is more significant.

**Figure 6:**
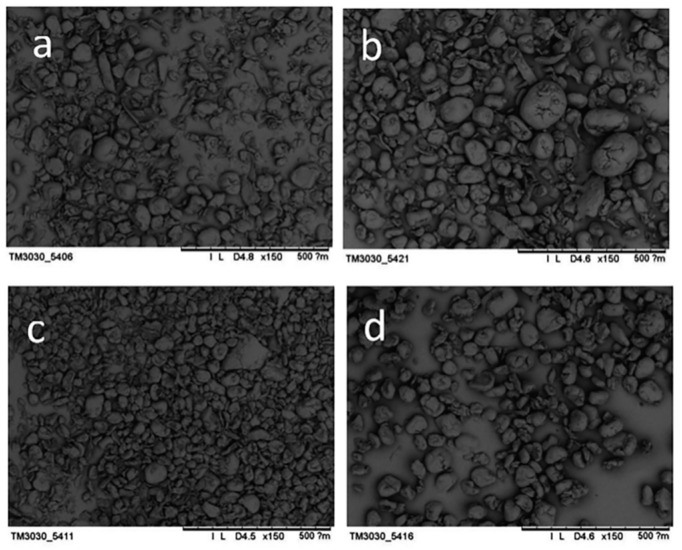
Morphological changes of PET particles before and after whole-cell biocatalysis treatment. (**a**) PET sample before treatment; (**b**) PET substrate after alkali treatment; (**c**) PET substrate after whole cell biocatalysis under alkaline conditions; (**d**) PET substrate after whole cell biocatalysis under neutral conditions.

In order to further study the whole-cell catalyzed PET biodegradation under alkaline conditions, the morphological and structural changes of PET particles before and after biodegradation were observed. The results are shown in Figure 6.

It can be seen from Figure 6 that PET particles have a wide range of particle distribution before processing, and there are irregular forms of PET such as flakes, ellipses and rods, which are caused by the inherent defects of mechanical pulverization in the physical grinding process; the main particle size is concentrated and the average particle size is basically consistent with the average particle size of the laser scattering particle size analyzer. The morphology of PET substrate samples after chemical degradation, biocatalytic degradation and biochemical combination catalytic decomposition showed nearly spherical particles whose surface was glossy, and the particle size distribution was narrow. By comparison, it was found that the alkali-treated PET had a wide particle size distribution and uneven size; while the PET substrate after bioprocessing of PET decomposing bacteria F4 had a narrow particle size distribution and a uniform particle size and the PET substrate has a narrower particle size distribution and a more uniform particle size after catalytic decomposition by the alkali-resistant PET decomposition bacteria. Comparing the average particle diameters from large to small is: a > b > d > c, which is consistent with the above-mentioned laser particle size scatterometer (ZetaSizer Nano-Series).

#### 3.3.2. Changes in PET Supramolecular Structure

**Figure 7:**
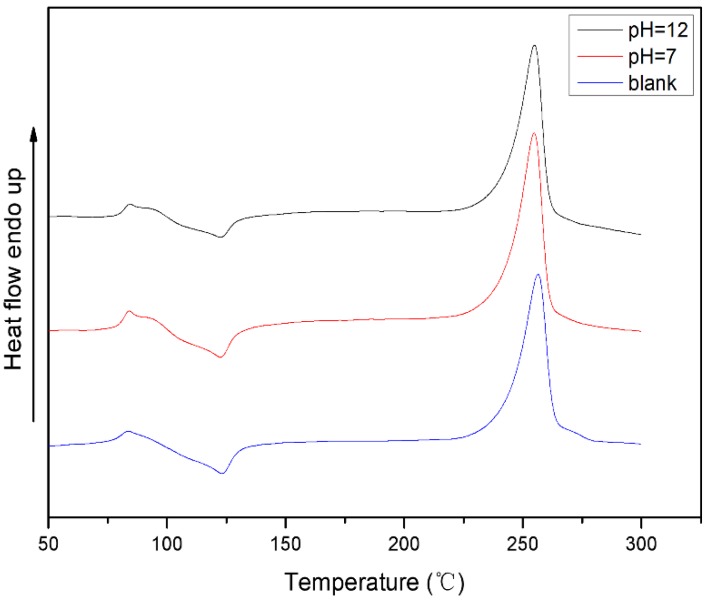
Thermal properties of PET substrates after whole cell biological treatment under different pH conditions.

In order to study the supramolecular structure changes of PET substrate after biocatalytic decomposition of whole cells, the thermal properties of PET particles after biodegradation treatment at different pH were examined. The results are shown in Figure 7.

The DSC test results showed that the crystallinity of the PET samples increased after biological treatment under pH = 12 and pH = 7. This indicates that areas of low crystallinity or amorphous areas in the PET substrate are more susceptible to biodegradation, leaving relatively high crystallinity. This has been proposed in the study of degradation of PET membranes by enzymes.

**Figure 8:**
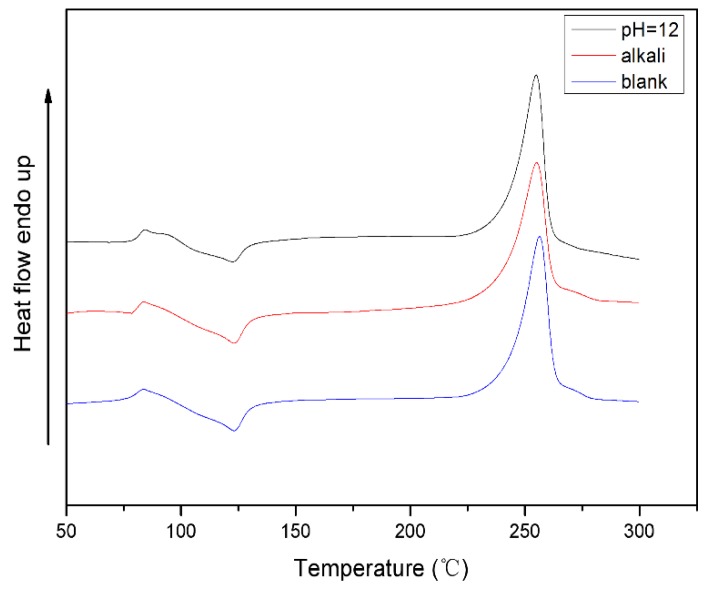
Differential scanning calorimetry thermogram analysis of PET treated by alkali-resistant strains and PET treated by alkalis.

Whole-cell biodegradation of PET under alkaline conditions includes both biocatalytic and base catalyzed hydrolysis. In order to further understand the effect of whole-cell biodegradation on the thermal properties of PET substrates under alkaline conditions, the PET particles were separately treated with alkali-resistant strains and alkali and the results are shown in Figure 8.

The melting point (*T*_m_), the glass transition temperature (*T*_g_), the enthalpy change (Δ*H*_c_) of the crystallization peak, and the enthalpy change (Δ*H*_m_) of the PET obtained from the DSC curve are listed in Table 3.

It can be seen from Table 3 that the crystallinity of PET after different treatments is improved, wherein the increase in crystallinity of PET treated with alkali-resistant PET decomposing bacteria is 1.50%, and the crystallinity of PET treated with alkali chemical treatment is increased by 0.88%. Therefore, the crystallinity of PET is improved and the crystallinity of PET after alkaline biodegradation is increased the most. The results further indicate that the biodegradation of PET under alkaline conditions is not only the hydrolysis under the action of alkali, but the result of the combination of biodegradation and alkali hydrolysis.

The high degree of crystallinity means the dense structure of the PET, which is an important reason why PET is difficult to biodegrade. As a material that is difficult to biodegrade, the fragmentation decomposition from the surface in PET biodegradation process is the bottleneck of the whole process and the least efficient step in the whole process. The hydrolysis of PET surface caused by base catalysis is beneficial to break through the barrier of PET biodegradation. Accelerate the formation of small molecule decomposition products in the fermentation broth so that they can be absorbed by the cells as a carbon source more quickly, thereby contributing to the growth of strains. In the system of whole cell biocatalytic degradation of PET under alkaline conditions, the chemical catalyst and the biocatalyst can alternate and complement each other to form a virtuous cycle. Before the strain enters the logarithmic growth phase, the amount of PET degrading enzyme is small and the activity is low while PET is partially decomposed under the condition of alkali catalysis, and the small molecular substance such as TA formed which can be utilized as a carbon source by the strain; As the strain enters the log phase, the amount and activity of the secreted extracellular enzymes increase, the decomposition of PET under biocatalysis accelerates, and the decomposition products in the system are more. The TA, one of the main acidic products, can be neutralized by the base in the system, avoiding the inhibition of strain growth and enzyme function. At the same time, the system gradually changes from a higher pH to a more neutral, which is more conducive to the growth and metabolism of the strain and the production of enzymes. Moreover, products such as TA in the system can be utilized by microorganisms.

Therefore, for PET, which is recalcitrant to biological degradation, the combination of biological treatment and chemical treatment helps to break the barrier structure of bioprocess resistance and improve the efficiency of biological degradation.

## 4. Conclusions

Compared with enzymes, biodegradation of PET by whole-cell catalyst have more complicated products. In case of bacterial whole-cell biodegradation of PET, the products can be used by strain growth and do not accumulated in culture solution. Thus, feedback inhibition of products can be avoided. The change of PET particle after biodegradation indicated that this biodecomposition is a layer-by-layer decomposition process from outside to inside.

When PET was treated with an alkaline strain under high pH conditions, the product concentration was higher than that of the biocatalyst under neutral conditions. Comparison with the neutral conditions, the PET decomposition strain under alkaline conditions grew faster and the total biomass was higher; the PET particles decreased more and the crystallinity changed more. This shows that the method of combined processing of alkali and organisms is more efficient for biodegradation of PET.

The combination of biological treatment and chemical treatment helps to break the resistance barrier structure of its bioprocessing and improve the efficiency of biodegradation. This method of combined processing, which is more industrialized and applied, is expected to be used to solve the pollution of micro plastics.

## Figures and Tables

**Table 1:** Culture composition of bacteria F4.

Reagent	Concentration (g/L)	Reagent	Concentration (μg/L)
NH_4_Cl	1.00	CuSO_4_·5H_2_O	40.0
KH_2_PO_4_	3.00	FeCl_3_·6H_2_O	0.2
Na_2_HPO_4_	7.00	MnSO_4_·5H_2_O	0.4
NaCl	0.50	ZnCl_2_	0.4
MgSO_4_·7H_2_O	0.25	(NH_4_)_6_Mo_7_O_24_·7H_2_O	0.2
PET	1	H_3_BO_3_	0.5

**Table 2:** Culture composition of bacteria F6.

Reagent	Concentration (g/L)	Reagent	Concentration (μg/L)
NH_4_Cl	1.0	H_3_BO_3_	0.5
NaOH	1.4	FeCl_3_·6H_2_O	0.2
Kcl	3.7	MnSO_4_·5H_2_O	0.4
NaCl	0.5	ZnCl_2_	0.4
MgSO_4_·7H_2_O	0.25	CuSO_4_·5H_2_O	40
PET	1	(NH4)_6_Mo_7_O_24_·7H_2_O	0.2

**Table 3:** Thermodynamic properties of PET samples.

Sample	*T*_m_	*T*_g_	Δ*H*_c_	Δ*H*_m_	Crystallinity
	°C	°C	J·g^−1^	J·g^−1^	(%)
①	256.4	81.2	−16.28 (123.0 °C)	48.51	23.02
②	255.1	81.6	−16.51 (122.4 °C)	50.08	23.98
③	254.9	81.9	−14.03 (122.4 °C)	48.36	24.52

① PET; ② PET treated by alkali; ③ PET treated by alkali bacterial.
